# Vitamin E inhibits the UVAI induction of “light” and “dark” cyclobutane pyrimidine dimers, and oxidatively generated DNA damage, in keratinocytes

**DOI:** 10.1038/s41598-017-18924-4

**Published:** 2018-01-11

**Authors:** George J. Delinasios, Mahsa Karbaschi, Marcus S. Cooke, Antony R. Young

**Affiliations:** 1 King’s College London, St John’s Institute of Dermatology, 9th Floor, Tower Wing, Guy’s Hospital; Great Maze Pond, London, SE1 9RT UK; 2 Oxidative Stress Group, Department of Cancer Studies, University Hospitals of Leicester NHS Trust, Leicester, UK; 3 Department of Genetics, University of Leicester, Leicester Royal Infirmary, University Hospitals of Leicester NHS Trust, Leicester, UK; 4 Present Address: Oxidative Stress Group, Department of Environmental Health Sciences; and Biomolecular Sciences Institute, Florida International University, University Park, 11200 SW 8th Street, Miami, Fl 33199 USA; 5 Present Address: International Institute of Anticancer Research, Kapandriti, 19014 Greece

**Keywords:** DNA, DNA adducts

## Abstract

Solar ultraviolet radiation (UVR)-induced DNA damage has acute, and long-term adverse effects in the skin. This damage arises directly by absorption of UVR, and indirectly *via* photosensitization reactions. The aim of the present study was to assess the effects of vitamin E on UVAI-induced DNA damage in keratinocytes *in vitro*. Incubation with vitamin E before UVAI exposure decreased the formation of oxidized purines (with a decrease in intracellular oxidizing species), and cyclobutane pyrimidine dimers (CPD). A possible sunscreening effect was excluded when similar results were obtained following vitamin E addition after UVAI exposure. Our data showed that DNA damage by UVA-induced photosensitization reactions can be inhibited by the introduction of vitamin E either pre- or post-irradiation, for both oxidized purines and CPD (including so-called “dark” CPDs). These data validate the evidence that some CPD are induced by UVAI initially *via* photosensitization, and some *via* chemoexcitation, and support the evidence that vitamin E can intervene in this pathway to prevent CPD formation in keratinocytes. We propose the inclusion of similar agents into topical sunscreens and aftersun preparations which, for the latter in particular, represents a means to mitigate on-going DNA damage formation, even after sun exposure has ended.

## Introduction

The short- and long-term consequences of solar UVR (~295–400 nm) exposure are well-established^1^ but detailed knowledge of the, spectral effects and mechanisms, especially long-term effects, is lacking. The majority (>95%) of solar UVR is UVA, most of which (~75%) is UVAI (340–400 nm). This spectral region penetrates the skin deeper that UVB (280–320 nm), readily reaching the dermal collagen and elastic fibres^2^.

The mutagenicity of UVA is caused through induction of DNA damage *via* direct absorption of UVR by DNA, and indirectly *via* photosensitization reactions^3,4^. This mutagenicity has been attributed, at least in part, to oxidatively generated modification of DNA nucleobases^5^. One of the most intensively studied lesions is the oxidatively modified purine, 8-oxo-7,8-dihydroguanine (8-oxoGua). This lesion is a possible contributor to UVA mutagenesis, and its presence has been studied in both epidermal DNA and urine^6–8^. It has also been proposed that UVA may increase intracellular oxidative stress without the generation of additional reactive oxygen species (ROS), by increasing the ratio of GSSG/GSH^9^. Cyclobutane pyrimidine dimers (CPDs) are DNA photolesions that have important biological consequences, including mutagenicity, which may lead to keratinocyte cancers of the skin^10^. CPDs also have non-mutagenic consequences such as initiating cytokine release^11^, and photoimmunosuppression that are also thought to be involved in skin cancer^12^. Importantly, a recent report has demonstrated that UVA exposure of melanin can trigger the formation of CPD via chemically generated, excited electronic states. The resulting so-called “dark” CPD, can continue to be formed for at least 3 h after UVA exposure^13^, in contrast to CPD formed immediately upon irradiation (now considered “light” CPD). This phenomenon has been demonstrated previously in melanocytes, and melanosome recipient keratinocytes *in vivo*, but not human keratinocytes.

The increasing incidence of skin cancer in sun-sensitive, white-skinned populations has initiated great debate on acute and long-term photoprotection. Sunscreens have limitations^14^ and consequently other methods of photoprotection are being sought. These include the use of various antioxidant substances, although it has often been difficult to prove their efficacy for protection of human skin *in vivo*.

α-Tocopherol (vitamin E) is a well-known antioxidant that is believed to be the most important naturally occurring non-enzymatic, lipid-soluble antioxidant in human tissue. Vitamin E can scavenge UVA-induced free radicals, protect endogenous epidermal antioxidant degradation and prevent lipid peroxidation, as well as inhibit UVR-induced immunosuppression^15–17^. Vitamin E has been employed in combination with vitamin C revealing significant protection against sunburn and erythema, indicating potential protection against skin cancer and photoageing^18,19^. Apart from its free radical scavenging properties, the application of vitamin E prior to UVR exposure has attracted attention for its ability to prevent the formation of UVB-induced CPD^20^. This property has been reported in mouse skin *in vivo* (samples were obtained immediately after UVR), however it is unclear as to whether this should be attributed to a sunscreen effect or some other activity of vitamin E^21–23^, the precise nature of which is unclear.

Despite the generally accepted beneficial effects of vitamin E, its photoprotective properties, especially on human skin cells, against UVA and UVB irradiation have not been clearly established. The current study was undertaken in order to determine the potential for vitamin E to protect against UVAI-induced photolesions, with particular emphasis on dark CPDs, in keratinocytes.

## Results

### Pre-UVAI treatment with vitamin E protects against oxidizing species and DNA damage

**Figure 1:**
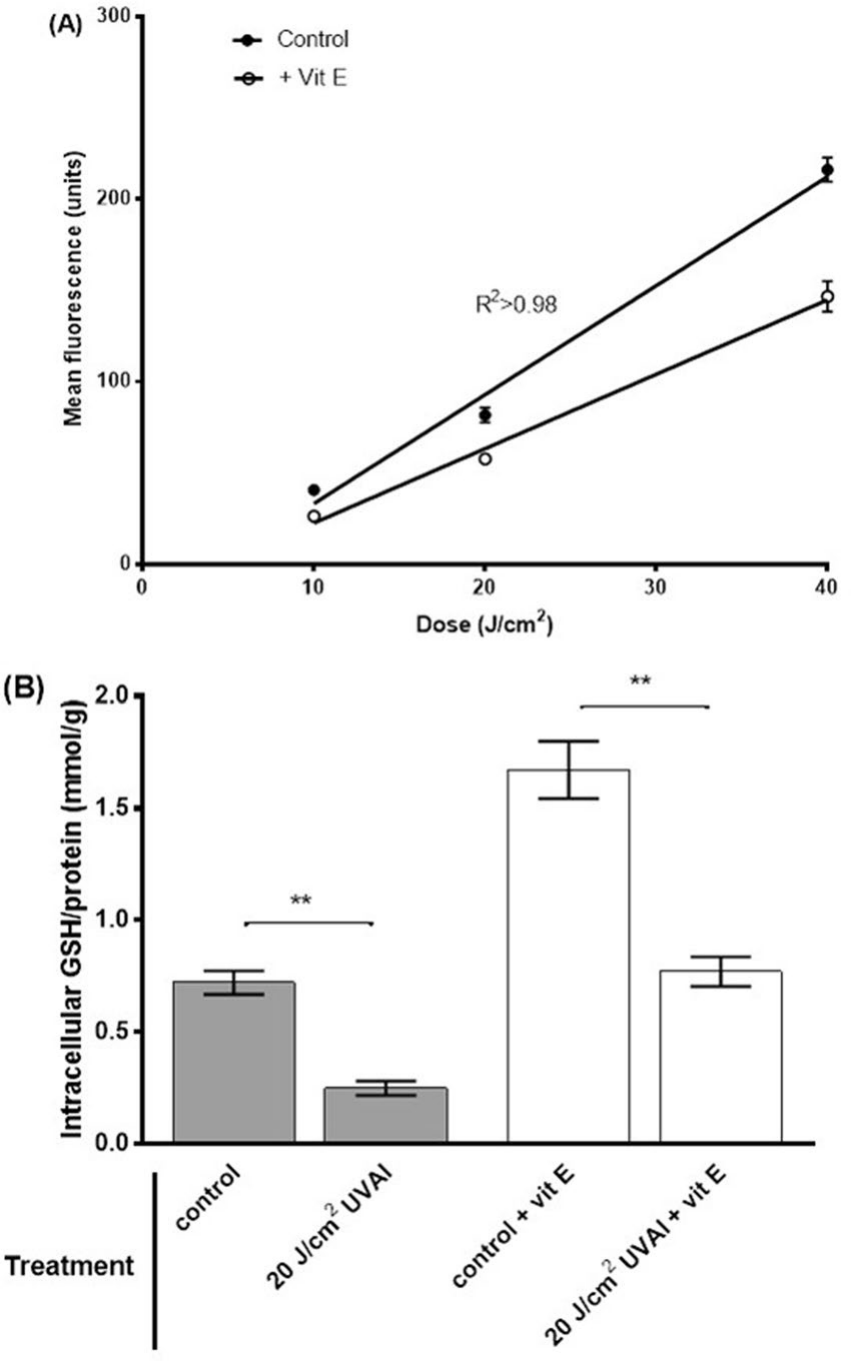
(**A**) UVAI-induced oxidizing species dose-response in: control (EtOH) or vitamin E (+Vit E) pre-treated groups; cells were treated with vitamin E for 24 h followed by UVAI irradiation. The production of oxidising species was determined by H_2_DCFDA fluorescence, coupled with flow cytometry. Results represent the mean of three independent experiments ±SEM. The UVAI dose-responses were determined by linear regression analyses. R^2^ was >0.98 and slopes were very significantly different from zero (p < 0.0001). (**B**) Effect of vitamin E supplementation on intracellular HaCaT GSH levels. HaCaTs were supplemented with vitamin E for 24 h prior to UVAI irradiation. Data are expressed as the means ± SEM of three independent experiments. **p < 0.01.

Cell viability was found to be unaffected by UVAI exposure and/or vitamin E treatment (Table 1). A UVA dose-dependent increase in oxidizing species, determined by H_2_DCFDA fluorescence, was observed (Fig. 1A). Pre-UVAI treatment with vitamin E was found to offer significant protection at all UVAI doses tested. This was demonstrated at all cases (p < 0.05), with the effect being more evident at higher UVAI doses. The effect was highly significant at 40 J/cm^2^ with a 35% decrease in oxidizing species compared to control (p < 0.001). Vitamin E did not alter the level of intracellular oxidizing species in unirradiated cells. Incubating HaCaTs with vitamin E for 24 h, prior to irradiation, significantly increased intracellular GSH levels by 2.3-fold (p = 0.002), and protected against UVAI-induced GSH depletion (Fig. 1B), suggesting that the UVAI-induced oxidizing species detected above are, at least in part, ROS.

**Table 1:** HaCaT cell viability following UVA ± vit E. Cell viability was assessed by the trypan blue (TB) exclusion and the MTT assays.

Treatment	Viable cells (%) TB	Viable cells (%) MTT
Control*	87.3 ± 3.2	88.7 ± 2.5
20 J/cm^2^ UVAI*	81.8 ± 4.3	80.1 ± 3.6
Vitamin E	88.6 ± 2.5	89.2 ± 2.0
20 J/cm^2^ UVA + Vitamin E	85.9 ± 1.6	86.5 ± 2.5

Data represent the mean of three independent experiments ± SEM. One-way ANOVA with Bonferroni correction showed significant differences between all groups (p < 0.05). *Cells incubated with EtOH for 24 h.

**Figure 2:**
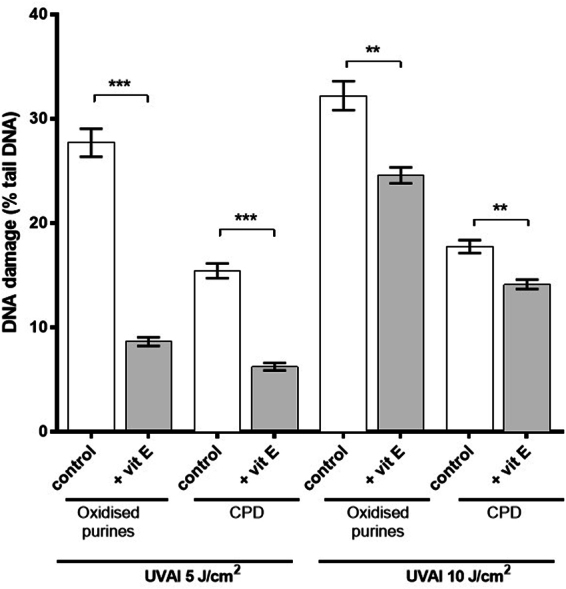
Effect of pre-UVAI incubation with vitamin E on the formation of oxidized purines and CPD. Mean percentage of tail DNA was determined following UVAI doses of 5 and 10 J/cm^2^. Results are the mean ± SEM of three independent experiments; ***p < 0.001, **p < 0.01 for selected comparisons.

Comet analysis in the absence of any enzyme treatment assesses alkali labile sites (ALS) and frank strand breaks (SB). Throughout, the comet assay data for oxidized purines and CPD formation were corrected for the levels of ALS and SB (ALS/SB background values were subtracted from the corresponding hOGG1- and T4endoV-derived values). A UVAI dose-dependent increase in both oxidized purines and CPD formation was demonstrated (Fig. 2). Pre-irradiation treatment of HaCaTs with vitamin E offered a statistically significant protection against UVAI-induced oxidized purines. This effect was observed at both UVAI doses tested (5 and 10 J/cm^2^), and was more evident at 5 J/cm^2^ (66% decrease; p < 0.001). Interestingly, pre-UVA vitamin E treatment was found to also inhibit UVAI-induced CPD formation, at both UVAI doses tested (60% and 23% decrease at 5 and 10 J/cm^2^, with p < 0.001 and p < 0.01, respectively).

### Post-UVAI treatment with vitamin E protects against oxidizing species and DNA damage

**Figure 3:**
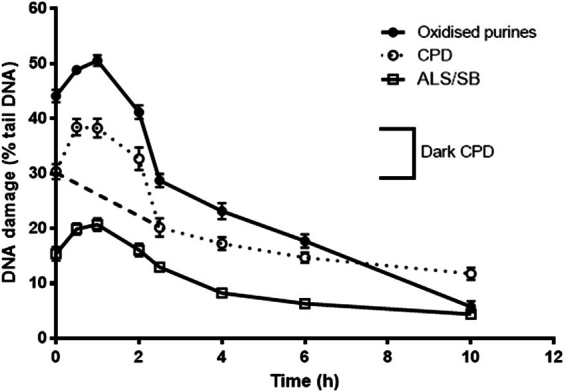
Induction and repair of UVAI-induced oxidized purines and CPDs, determined by the T4endoV- and hOGG1-modified comet assay. HaCaT keratinocytes were irradiated with 5 J/cm^2^ UVA and were left to repair for different time periods. At 0 h, the dotted line mainly represents the formation of “light” CPD. The subsequent increase, with a peak at 1 h, represents the formation of “dark” CPD and their repair (1–2.5 h). The dashed line, which joins the dotted line, represents the proposed, differential repair of “light” CPD. The results are the mean ± SEM of three independent experiments.

**Figure 4:**
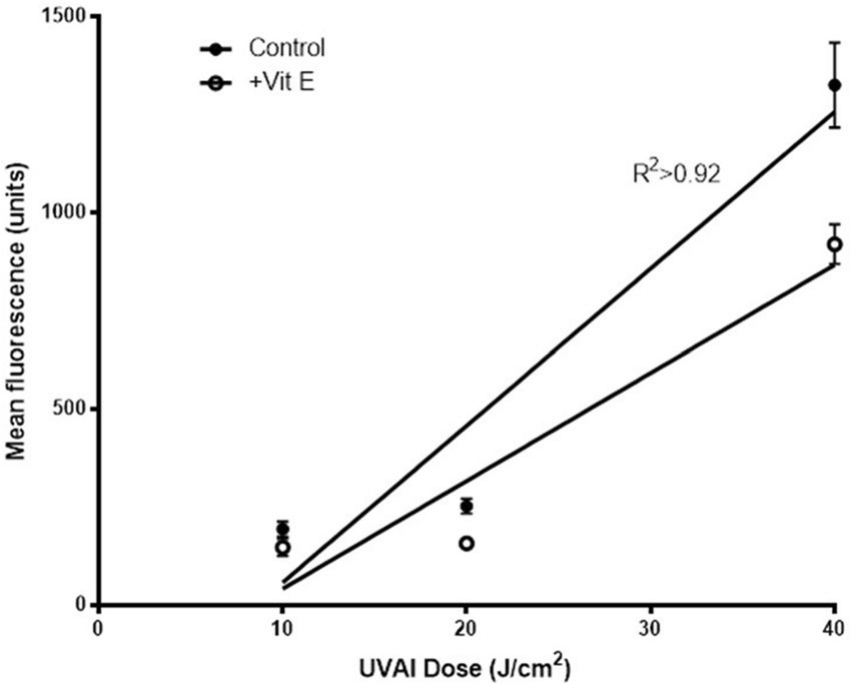
UVAI-induced oxidizing species dose-response in: control (EtOH) or vitamin E (+Vit E) treated groups; cells were treated with vitamin E for 2.5 h, after UVA irradiation. Production of oxidising species was determined by H_2_DCFDA fluorescence. Results represent the mean of three independent experiments ± SEM. The UVA dose-responses were determined by linear regression analyses. R^2^ was >0.92 and slopes were very significantly different from zero (p < 0.0001).

**Figure 5:**
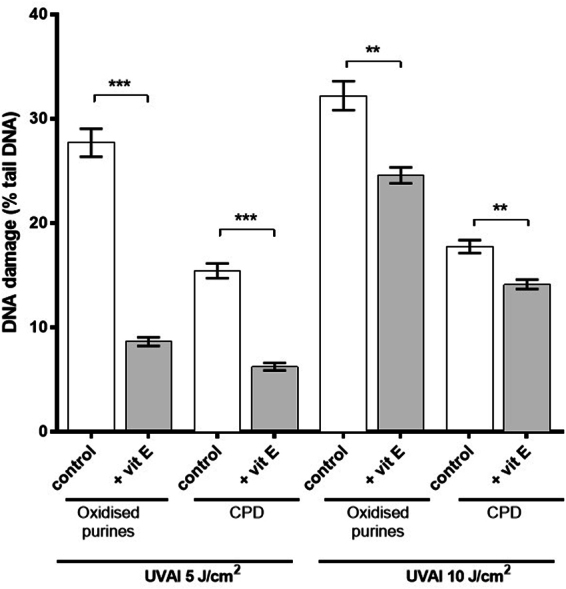
Effects of post-UVAI incubation on oxidized purine and CPD formation. Mean percentage of tail DNA was determined following UVA doses of 5 and 10 J/cm^2^ and treatment of cells with vitamin E for 2.5 h. Results are the mean ± SEM of three independent experiments; ***p < 0.001, **p < 0.01, *p < 0.05 for selected comparisons.

A time-course study showed that all DNA lesions increased after UVAI exposure (0 h) with a peak of formation at 1 h (Fig. 3). This was partly expected for oxidized purines and ALS/SB, due to their formation by reactive intermediates, but was surprising for CPD. This reveals the delayed induction of dark CPD in keratinocytes, even after removal from UVAI exposure (dotted line), and then their repair, contrasting with the expected repair of CPD (dashed line; Fig. 3). The increase and decrease in CPD levels during the 0–2.5 h period was the basis for determining the time for the post-UVA vitamin E incubations in subsequent studies (*i.e*. Figs 4 and 5).

Cells treated with vitamin E exhibited lower levels of oxidizing species compared to their untreated counterparts (Fig. 4). This clear protective effect of vitamin E was found to be greater following doses of 20 and 40 J/cm^2^ (maximal inhibition of the production of oxidizing species was 38%, at 40 J/cm^2^; p < 0.05). The post-UVA protective effect of vitamin E was also evident on DNA damage. Vitamin E treatment significantly decreased the formation of oxidized purines, compared to non-treated cells (70% and 32% decreases at 5 and 10 J/cm^2^, with p < 0.001 and p < 0.01, respectively) (Fig. 5). Post-UVA vitamin E treatment was also found to decrease CPD formation, an observation that cannot be attributed to a possible sunscreen property, since vitamin E was added after UVA exposure. CPD values were decreased at both UVA doses tested (52% and 44% decrease at 5 and 10 J/cm^2^, with p < 0.05 and p < 0.01, respectively). Induced DNA damage was lower in the post-UVAI incubation control experiments compared to incubation pre-UVAI, especially at 5 J/cm^2^. This suggests DNA repair occurs within 2.5 h of exposure.

### Vitamin E prevents UVC-induced formation of oxidized purines, but not CPD

**Figure 6:**
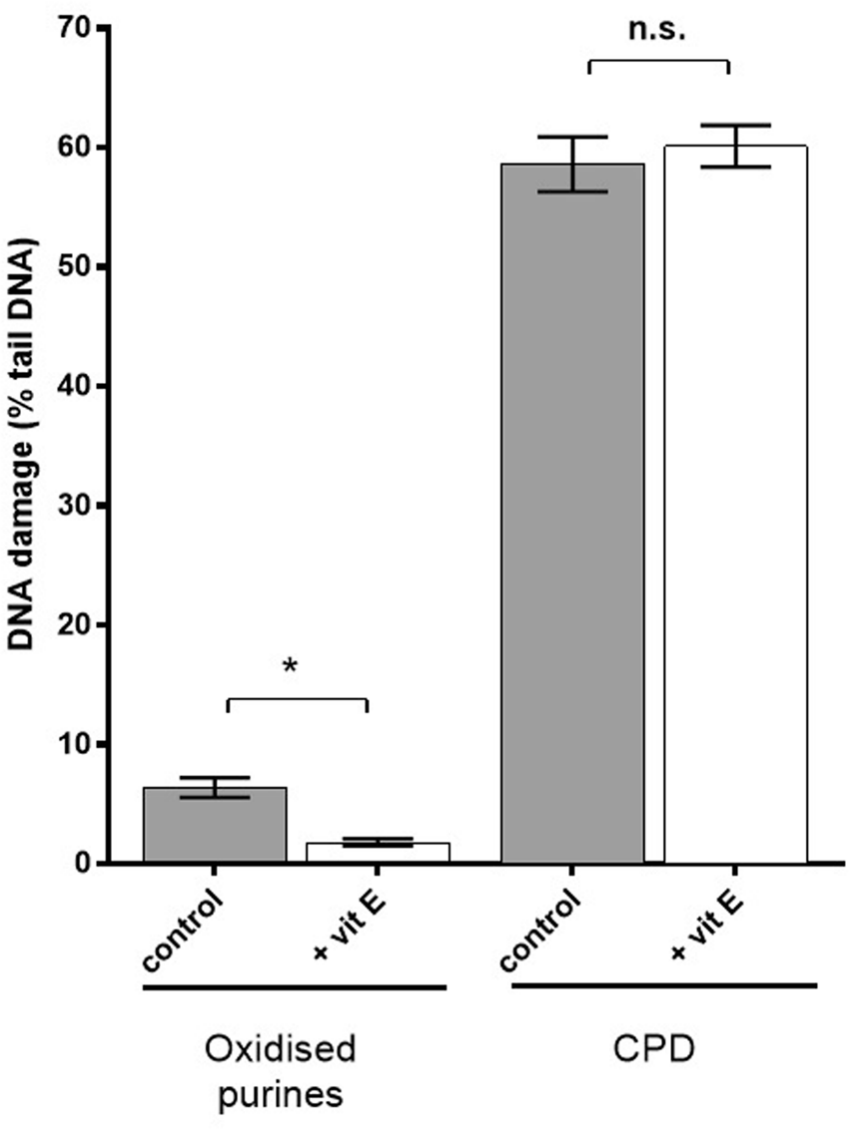
Effect of vitamin E pre-incubation on UVC-induced formation of CPD and oxidized purines, determined by T4endoV- and hOGG1-modified comet assays, respectively. The results represent mean (±SEM) percentage tail DNA in HaCaT cells exposed to UVC for 10 s, and have been corrected for baseline levels of damage and SB/ALS. Results are the mean ± SEM of three independent experiments; *p < 0.05 for selected comparisons.

Monochromatic UVC (254 nm) was employed to assess a possible sunscreening role of vitamin E (see discussion section for fuller rationale). As expected; UVC induced high CPD levels, together with some oxidized purines, ALS and SB. Incubation with vitamin E pre-UVC led to a significant decrease (Fig. 6) in ALS and SB and oxidized purines (66%, p < 0.05) but had no effect on CPD formation.

## Discussion

UVAI is by far the major spectral component of solar UVR and penetrates deeper into the skin than UVB. A study of DNA damage depth profiles in human skin *in vivo* shows attenuation of CPD and pyrimidine (6–4) pyrimidone photoproduct formation with increasing with skin (epidermis and dermis) depth, but the reverse is true, for CPD at least, with UVAI^24^. This results in greater sensitivity of the keratinocyte stem cell and melanocyte containing basal layer to UVAI exposure. UVA-induced mutations are more prevalent in the basal layer than the supra-basal layers^25^. It is therefore important to find new strategies to protect the skin, especially the basal layer, from UVAI-induced DNA damage. Vitamin E has been established as a UVR-induced ROS scavenger. To the best of our knowledge, we provide the first evidence that UVAI-induced CPD (including dark CPD), as well as oxidatively-generated DNA lesions, can be inhibited by vitamin E in HaCaT keratinocytes. Whilst the latter is in line with its classical role as an antioxidant, the former is an important finding, given earlier, similar findings, but in melanocytes^13^. Our UVAI doses (5 and 10 J/cm^2^) were sub-erythemal and physiologically and environmentally relevant; a minimal erythema dose (MED) of UVAI is about 50 J/cm^2^ in fair skin types^26^.

Carboxy-H_2_DCFDA was selected to study the formation of oxidizing species because it can be oxidized by several UVA-induced reactive oxygen species and free radicals (including H_2_O_2_, NO and peroxides)^27^. The data showed a UVAI dose-dependent increase in oxidizing species formation in HaCaT cells. Cells treated with vitamin E, prior to UVA, produced less oxidizing species compared to controls at all tested doses. Under these same conditions, levels of intracellular GSH increased, and subsequently provided some protection against the UVAI induction of oxidizing species suggesting that at least some of these oxidizing species are ROS.

Interestingly, a protective effect was also seen when vitamin E was administered post-UVA. It is noteworthy that more oxidizing species were generated post- versus pre-UVA incubation. This observation may be attributed to the formation of oxidizing species *via* secondary biochemical pathways^28^. These species can result from lipid peroxidation (which can be inhibited by vitamin E), which in turn might initiate the formation of further oxidatively damaged DNA.

The hOGG1-modified comet assay showed that UVAI induced the formation of oxidized purines in a dose-dependent manner. Vitamin E offered a significant protective effect with pre- and post-exposure incubation. Since the UVA-induced oxidation of purines are formed indirectly, predominantly *via* the photosensitizer-dependent induction of ^1^O_2_^29,30^, the antioxidant/scavenging properties of vitamin E were not surprising, although this protective effect has not previously been demonstrated for oxidized purines by the comet assay, especially with post-UVAI incubation. This suggests that, after the initial ROS generation via photochemical processes (energy transfer to molecular oxygen, electron abstraction, etc), biochemical pathways are then responsible for the generation of secondary ROS, possibly through ^1^O_2_ production^31,32^. This may occur *via* UVA-induced enzyme activity, e.g. activation of NADPH oxidase^28^. NADPH oxidase increases UVA-induced superoxide, in mouse, monkey and human cell lines^33^, which can be converted to other ROS. Other studies show evidence of a protective effect of vitamin E against ovulation-induced 8-oxoGua in ovarian epithelial cells^34^, as well as ozone-induced 8-oxoGua^35^. The antioxidant role of vitamin E has been reported to protect against *cis*-urocanic acid-induced ROS^36^. It is worth noting that ^1^O_2_ does not generate strand breaks, although some alkali-labile sites (both of which may be evaluated by the comet assay) can be produced (as noted in Cooke *et al*.^37^, but predominantly it is the nucleobase modification, 8-oxoGua, that is generated^38^.

**Figure 7:**
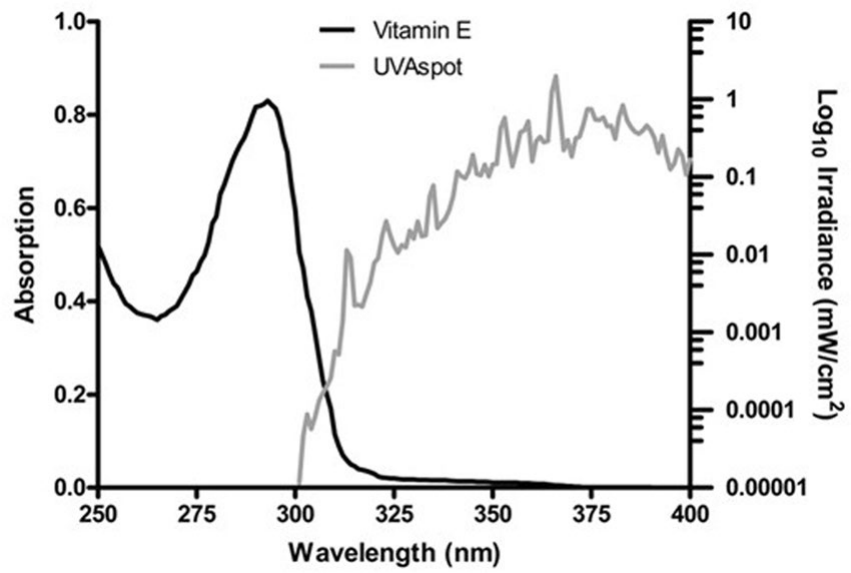
Emission spectrum of the “UVA spot”. This was determined by a Bentham DM150 double monochromator spectroradiometer through the plastic lid of a petri dish in which cells were irradiated, at a distance of 39 cm. Also shown is the absorption spectrum of a vitamin E solution (0.1 mg/mL in ethanol).

CPDs, assessed by the T4endoV-modified comet assay, were readily induced by UVAI irradiation, and their formation was significantly inhibited by incubation with vitamin E before and after irradiation. The ability of post-UVAI vitamin E incubation to inhibit CPD excludes a sunscreening effect, but also alludes to an indirect mechanism for UVAI-induced CPD. This is supported by the irradiation studies with UVC (254 nm), which is close to the action spectrum maximum for CPD induction *in vitro*^39^. Action spectroscopy shows that production of CPD at 300–310 nm is three orders of magnitude lower than at 254 nm^40^. Figure 6 shows that 254 nm induced high levels of CPD as well as oxidized purines. Although UVC-induced 8-oxoGua has been previously reported^41–43^ there is little literature on UVC-induced cellular oxidative stress. One report has suggested that UVC-induced 8-oxoGua formation is *via* ^1^O_2_^30^, although it is not clear how this would occur, and the involvement of guanine radical cations are perhaps a more likely mechanism^44^. Pre-UVC incubation with vitamin E significantly protected against the formation of oxidized purines but not against CPD (Fig. 6). As can be seen in Fig. 7, vitamin E absorption at 254 and ~300 nm is similar, but higher than at longer wavelengths in the UVAI source. Thus, one might expect a comparable or better sunscreening effect for CPD at 254 nm than with UVAI. The lack of such an effect for CPD suggests that protection against the formation of oxidized purines is via mechanisms other than sunscreening. Furthermore, the action spectrum for 8-oxoGua formation shows a peak in the UVAI (λ_max_ = 365 nm) region^40^, although UVB may also induce this lesion^7^. We recognize that the very small UVB content (0.1%) of our UVAI source (Table 2) may have caused a disproportionally large number of CPD^45^ but the complete lack of effect of vitamin E on UVC-induced CPD supports a different mechanism for UVAI-induced CPD.

**Table 2:** Spectroradiometric distribution of the emission spectrum (see Fig. 1) of the UVAspot (measured through the plastic lid of a 6-well plate used in the experiments), and their respective erythemally effective energies (EEE), obtained by multiplying with the CIE action spectrum for erythema^25^.

Spectral Region	Wavelength (nm)	% of total irradiance	% EEE
**UVA**	**321–400**	**99.8**	**87.5**
*UVAI*	*340–400*	*97.5*	*78.4*
*UVAII*	*321–340*	*2.3*	*9.1*
UVB (CIE)*	**281–315**	**0.1**	**10.4**
**UVB**	**281–320**	**0.2**	**12.7**
**UVC**	**200–280**	**0.0**	**0**
**Total UVR**	**200–400**	**100**	**100**

This shows that the majority of the EEE was in the UVAI region. *Official CIE definition of UVB, but a cut-off at 320 nm is used in dermatology research.

CPDs are formed mainly *via* direct photon absorption by DNA in the UVC and UVB regions. DNA shows some direct UVA absorption^46^ which is well established to result in CPD formation *in vitro* and *in vivo*, in cellular^47–51^, and naked DNA^4,51–56^, and is likely to represent the main pathway for the majority of CPD formation, with UVAI inducing CPD at a ratio of 87:9:4 for T <  > T, T <  > C and C <  > C, respectively^50^. However, UVA-induced CPD may also be formed by photosensitization, and chemiexcitation reactions *in vitro* as with carprofen^57^ and melanin^13^. In this case the photosensitizer is converted to its triplet state, the energy of which, if high enough, is transferred to DNA to generate CPD^58^. Therefore, unidentified endogenous photosensitizers, which we have proposed may possess structural similarities to pyridopsoralens^59,60^, might play a role in UVAI-induced CPD formation in keratinocytes, especially in the more complex *in vivo* system. Our data suggest that vitamin E may quench the triplet state of a putative sensitizer. However, since similar levels of CPDs are induced by UVA in cellular and naked DNA, this^53,54,61^ suggests that triplet energy transfer may be a minor process. Our results in cultured keratinocytes indicate that there is an ~20% increase in CPD in the 1 h after removal from UVAI exposure.

UVA-induced lipofuscin in HaCaTs has been reported to be a photosensitizer for subsequent exposure (48 h later) to visible radiation, which results in the formation of oxidatively induced damage to DNA^62^. Although we did not measure visible radiation this was clearly present in our source (Fig. 7) especially in the violet region (400–450 nm), which is reported to be very effective in the generation of ^1^O_2_ from lipofuscin^63^. On this basis, it is possible that lipofuscin might be the unknown photosensitizer that is responsible for the ROS generation, and formation of oxidized purines and ALS in our study. However, we lack data on the lipofuscin triplet energy level so it is not possible to say whether or not lipofuscin could generate CPD by a sensitization reaction.

A comparison of the comet assay data from the pre- and post-UVA vitamin E incubation studies (Figs 2 and 5) suggests some repair of oxidized purines and CPD during the 2.5 h post-UVA period. This was confirmed in time-course studies with 5 J/cm^2^ (Fig. 3) that showed a t_1/2_ of about 4 h for both lesions (achieving a maximum at 1 h). This is similar to the t_1/2_ of ~4.5 h reported for UVA-induced CPD in HaCaTs in a recent publication from our group^64^. However, the t_1/2_ for oxidized purines in the current study is faster than that of about 10 h in our previous study, in which we also showed that DNA repair kinetics were dependent on UVR spectrum. The times for t_1/2_ are of course longer when using 0 h as a reference point. It should also be noted that very different UVA spectra were used in the two studies. Our present time-course study demonstrates the formation of “dark DNA photolesions”, that have been recently reported for CPD in melanocytes^13^ but not for oxidatively-induced DNA lesions.

It is possible that vitamin E incubation post-UVAI exposure decreases CPD by enhancing their repair by inhibiting ROS^65^ which, along with RNS, can damage/inhibit DNA repair enzymes and DNA polymerases (associated with both nucleotide excision repair (NER) and base excision repair (BER)^66,67^. This concept is supported by studies that show that post-UVA treatment with vitamin D_3_ suppresses nitric oxide products resulting in enhanced DNA repair; with a consequent reduction of CPD, immunosuppression and photocarcinogenesis^68^. UVA induces oxidatively-generated crosslinking (through ^1^O_2_ production) between the subunits of the replication and repair protein, PCNA^69^. Furthermore, UVA-induced ROS are also known to activate several MAPKs^70^, therefore, by affecting various downstream effectors, such as AP-1 and NFκB, they may alter DNA repair responses, cell cycle arrest or apoptosis^71^. One study showed that vitamin E treatment post-UVR (broad spectrum) irradiation increased CPD repair in mouse skin *in vivo* and this result was correlated with decreased p53 protein levels^72^.

In the present study, we showed that vitamin E can inhibit UVAI-induced oxidizing species production and induction of DNA damage, even when human keratinocytes are treated after irradiation. Whilst perhaps better established for ROS-induced DNA damage, the important implication of this is that the process of CPD formation continues after irradiation has ended, implying a mechanism similar to that seen in melanocytes^13,73^. Several questions remain about the chemistry behind the protection offered by vitamin E, and it is important to establish whether similar protection levels can be demonstrated in human skin.

An ideal sunscreen protects against UVR-induced, direct and indirect DNA damage, together with oxidative stress, and erythema. Our data, and those from other groups^4,54,74–76^, support the addition of antioxidants to sunscreens and after-sun preparations. Although relatively small, our demonstration of ‘dark’ CPD formation in keratinocytes, in the absence of melanin, indicates that such preparations should also contain agents which are both antioxidants and, for completeness, triplet state quenchers to decrease the formation of CPD in skin, which continues even after sunlight exposure has ended.

Figure 3 shows the induction and repair of CPD. The delayed, secondary increase, peaking at 0.5–1 h after the end of the UVR exposure, is very similar to that reported by Premi *et al*.^13^ for “dark” CPD. Whilst it is not possible to distinguish between ‘dark” CPD and the formation of those from direct UVR absorption (“light” CPD), we speculate that CPD burden at time 0 h primarily represents the latter and that the peak at 0.5–1 h represents the addition of “dark” to “light” CPD. We further speculate that there two overlapping CPD repair kinetics in Fig. 3. The dashed line from 0 to 2.5 h, and continuing to the dotted line, mainly reflect the typical NER kinetics of “light” CPD that is relatively slow with a half-life of 33.3 h in human epidermis *in vivo*^77^, but the rapid decline from the peak to the 3 h timepoint suggests a different, faster repair process for the “dark” CPD, more akin to the kinetics of the pyrimidine (6–4) pyrimidone photoproduct, with a half-life of 2.3 h in human epidermis *in vivo*^77^. Although fast repair of CPD, under particular conditions, is not without precedent^64^. The reasons for these differences remain to be elucidated. “light” and “dark” CPD may have different preferential nucleobases locations or properties that differentially activate NER. For example, “dark” CPD are reported to include a higher ratio of cytosine-containing (T <  > C and C <  > T), to thymine-thymine CPD^13^ and cytosine-containing CPD are more rapidly repaired^78^. It is clear that “dark” CPD remain an intriguing phenomenon, and about which there is no doubt much more for us to learn.

## Materials and Methods

### Cell culture and vitamin E treatment

The HaCaT cell line (spontaneously immortalized keratinocytes) was obtained from the American Type Culture Collection (ATCC, Manassas, VA, USA). Cells were cultured in Dulbecco’s modified Eagle’s medium (DMEM; Invitrogen, Paisley, UK) supplemented with 10% fetal calf serum (Sigma-Aldrich, Poole, UK), 100 U/mL penicillin, and 100 μg/mL streptomycin (Invitrogen) and maintained at 37 °C in 95% air/5% CO_2_. Vitamin E (D-α-tocopherol; Sigma-Aldrich, Poole, UK) concentration was fixed at 0.1 mM for all experiments. The experimental procedure was designed to include two treatment protocols. Cells were either treated with vitamin E for 24 h prior to UVA irradiation (pre-UVA treatment), or treated for 2.5 h after irradiation under the incubation conditions described above. Various post-UVA incubation times were tested in pilot studies and this time period was selected as the shortest period with a significant effect on both oxidized purines and CPD (data not shown).

### Irradiation and dosimetry

The UVAI source used was a UVASPOT (400/T, Dr K Hönle UVTechnologie, Munich, Germany), the spectrum of which is shown in Fig. 7, and described in Table 2. The erythemal effective energy (EEE) was calculated using the erythemal action spectrum of the International Commission on Illumination (CIE)^79^. Irradiance was determined with an International Light IL 442 A radiometer (Newbury Port, MA, USA) with a UVA detector calibrated against the measurements made with a double-monochromator spectroradiometer (Bentham Instruments, Reading, UK), which was calibrated against a UK national standard. Experiments to exclude any sunscreening effect of vitamin E were carried out with UVC (254 nm) using an XX-15s UV Bench Lamp (UVP, Cambridge, UK).

Cell irradiation (in monolayers) was performed at 17 cm from the UVAI source, in PBS and a maximum irradiation dose was fixed at 40 J/cm^2^. Comet assay experiments were accrued out with UVAI doses of 5 and 10 J/cm^2^, while for the oxidizing species detection assay doses of 10, 20 and 40 J/cm^2^ were used. As the UVAI source produced high levels of heat, a cooling platform was used at 6 °C that kept the cells at ~27 °C. Cells were kept on ice after irradiation/treatment before any processing. Control (unirradiated) cell cultures were maintained under the same conditions. UVC irradiation was performed at a distance of 25 cm from the lamp. Exposure was based on time (10 and 20 s), and determined empirically.

### Spectroscopy

UVR absorbance of vitamin E (0.1 mg/mL in ethanol) was determined with a UV/Vis Spectrophotometer (ATI *Unicam*, UK) between wavelengths 250–340 nm, to assess for a possible sunscreening effect. An overlap between the emission spectrum of the UVAspot and the vitamin E absorption spectrum can be observed in the 300–320 nm UVB region (Fig. 7).

### Cell viability

Twenty-four hours after UVAI exposure or vitamin E treatment, cell viability was determined using both the trypan blue exclusion assay, and the MTT assay, to ensure the absence of any significant cytotoxicity. Trypan blue (Sigma-Aldrich, Poole, UK) (0.04% final concentration) was added to cell suspensions and cells were counted in a haemacytometer. For the MTT assay, aliquots (20 μL) of MTT solution (10 mg/mL PBS) were added to the cells, and incubated for 4 h at 37 °C. After this, 100 μL of lysis solution (0.04 M HCl in absolute isopropanol) were added, and the cells shaken for approximately 10 min. The plates were subsequently read on a plate reader at 550 nm.

### Measurement of oxidizing species

We aimed to evaluate total intracellular ROS generation was detected using 5-(and-6)-carboxy-2′,7′-dichlorodihydrofluorescein diacetate (carboxy-H_2_DCFDA; Invitrogen, Paisley, UK). The reliability of this approach to measure intracellular H_2_O_2_ and ROS has been called into question, and a number of caveats need to be considered. Specifically, oxidizing species other than ROS may also oxidize carboxy-H_2_DCFDA to form a fluorescent product^80^ we acknowledge this caveat and use the term “oxidizing species” accordingly.

After UVAI exposure, 2 mL of 5 μM carboxy-H_2_DCFDA, diluted in PBS (containing 1 g/L glucose; Gibco) were added to cell suspensions (PBS-only was added in control samples). Cells were incubated for 20 min, in the dark, at 37 °C in a humidified incubator with 95% air/5% CO_2_. Plates were subsequently washed twice with PBS to completely remove any dye not internalized by the cells.

For the pre-UVR vitamin E incubation experiments, cells were analyzed immediately following irradiation. Following trypsinisation, cells were centrifuged at 400 × g for 4 min at 4 °C and resuspended in 0.5 mL of PBS + 0.1% (w/v) bovine serum albumin (BSA). Samples were then transferred to FACS tubes and analyzed with a Becton Dickinson FACSAria II instrument (BD Biosciences, San Jose, USA), using the FL1 channel (green fluorescence). The viable portion of the cell population was quantified by addition of 2.5 μg/mL propidium iodide (PI) immediately before the analysis. Cells were then subjected to analysis by flow cytometry.

### Total glutathione measurement

Intracellular concentrations of reduced glutathione (GSH) were determined using the GSH/GSSG kit (Calbiochem, La Jolla, CA,USA). Cell pellets were homogenized in 50 µL of cold metaphosphoric acid (5% w/v) and resuspended in a total volume of 500 µL. The homogenate was centrifuged for 10 min (3,000 × g) at 4 °C, before 100 µL of supernatant was combined with the kit, and analysed using a UV-vis spectrophotometer.

### Measurement of DNA damage by the comet assay

DNA damage was assessed using the alkaline comet assay with specific protocol modifications, according to the type of damage investigated. Oxidatively-induced DNA damage was measured using the human 8-oxoguanine DNA glycosylase 1 (hOGG1)-modified comet assay. hOGG1 recognizes 8-oxoGua, together with 2,6-diamino-4-hydroxy-5-formamidopyrimidine^81^, with minimal activity towards 4,6-diamino-5-formamidopyrimdine^82^. On this basis, and as previously^64^, we have used the term “oxidised purines” to describe the damage recognized by the hOGG1-modified comet assay. CPDs were assessed using the T4 endonuclease V (T4endoV)-modified comet assay, as described elsewhere^64^. As we have noted previously^64^, there are no specific data concerning the preferential activity of T4endoV towards the potential combinations of pyrimidines in CPD. However, inferences can be made from the ability of the enzyme to incise at all combinations of CPD, in plasmids and small bacteriophage vectors, suggesting all are equal substrates.

Cells were counted before UVR exposure and distributed in the wells of a 9-well plate (~10^3^ cells per well). After irradiation, cells were collected and centrifuged at 400 × g for 4 min, at 4 °C. The supernatant was discarded and pellets were kept on ice. Cells were mixed with 200 μL of low-melting point agarose and 75 μL of the gel was quickly poured onto slides pre-coated with agarose. Coverslips were placed over the gels, and the slides were kept on a tray on ice for 10 min to allow the agarose to set. Coverslips were then removed and the slides were placed in a tank filled with lysis buffer (2.5 M NaCl, 100 mM EDTA, 10 mM acid Tris, 1% sodium sarcosinate, pH 10, 1% Triton X-100, and 10% dimethyl sulphoxide) for 16 h at 4 °C, in the dark.

After removing the lysis buffer, slides were washed with ice-cold ddH_2_O for 10 min (in the dark, to prevent adventitious DNA damage). Slides were immersed twice in enzyme reaction buffer (40 mM Hepes, 0.1 M KCl, 0.5 mM EDTA, 0.2 mg/mL BSA, pH 8), for 5 min each, at room temperature. T4endoV (0.1 U/mL), or hOGG1 (3.2 U/mL), or enzyme reaction buffer alone, was added to each gel (both enzymes were purchased from New England Biolabs, Hitchin, UK). Coverslips were placed on top of the gels to ensure equal distribution of the enzymes, and slides were incubated at 37 °C in a humid atmosphere for 45 min. Slides were subsequently transferred to ice-cold electrophoresis buffer (NaOH 10 M, EDTA 200 mM, pH 13 in ddH_2_O) and incubated for 20 min in the dark. Electrophoresis was then performed for 20 min at 25 V, 300 mA.

Finally, slides were rinsed with neutralization solution (0.4 M Trizma Base, pH 7.5; Sigma) for 20 min and then washed with ddH_2_O for 10 min. Slides were allowed to dry at room temperature overnight. DNA was stained using 1 mL of propidium iodide solution at 2.5 μg/mL in PBS per slide for 20 min. Slides were then washed with ddH_2_O for 20 min. After drying, slides were examined at a magnification of 40×, using a Zeiss Axiophot epifluorescence microscope (Carl Zeiss, Germany) equipped with a green excitation filter. Images of the whole of each slide were taken with a Nikon camera linked to the microscope. Fifty randomly selected cells per gel and three gels per condition were analysed (n = 150) using Comet Score (TriTek Corp., Summerduck, VA, USA). The percentage tail DNA (%DNA) was measured for each nucleoid body scored.

**Figure 8:**
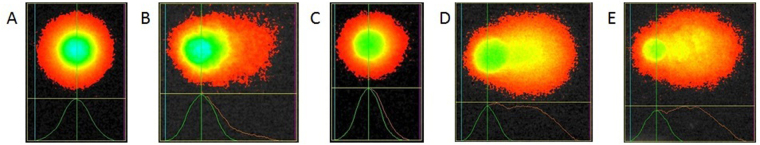
Representative images of (**A**) hOGG1-modified comet assay analysis of untreated cells; (**B**) hOGG1-modified comet assay analysis of cells treated with 10 J/cm^2^ UVA; (**C**) hOGG1-modified comet assay analysis of cells treated with 10 J/cm^2^ UVA, with post-irradiation with vitamin E; (**D**) T4endoV-modified comet assay analysis of cells treated with UVC for 10 s; and (**E**) T4endoV-modified comet assay analysis of cells treated with UVC for 10 s, after pre-incubation with vitamin E.

Representative images of comets are shown in Fig. 8.

### Statistical analysis

All experiments were conducted in triplicate and values are presented as mean ± standard error of the mean (SEM). Comet assay values were compared for statistical significance with the Mann–Whitney non-parametric test. For the measurement of oxidizing species, a Student’s t-test was used to determine the degree of statistical significance between values from different experimental groups; results were plotted. All analyses and graphs were performed using the GraphPad Prism 6 software (GraphPad Software Inc., San Diego, CA, USA). Statistical significance was defined as p < 0.05.
