# Author Correction: Vitamin E inhibits the UVAI induction of “light” and “dark” cyclobutane pyrimidine dimers, and oxidatively generated DNA damage, in keratinocytes

**DOI:** 10.1038/s41598-018-23048-4

**Published:** 2018-03-19

**Authors:** George J. Delinasios, Mahsa Karbaschi, Marcus S. Cooke, Antony R. Young

**Affiliations:** 1 King’s College London, St John’s Institute of Dermatology, 9th Floor, Tower Wing, Guy’s Hospital; Great Maze Pond, London, SE1 9RT UK; 2 Oxidative Stress Group, Department of Cancer Studies, University Hospitals of Leicester NHS Trust, Leicester, UK; 3 Department of Genetics, University of Leicester, Leicester Royal Infirmary, University Hospitals of Leicester NHS Trust, Leicester, UK; 4 Present Address: Oxidative Stress Group, Department of Environmental Health Sciences; and Biomolecular Sciences Institute, Florida International University, University Park, 11200 SW 8th Street, Miami, Fl 33199 USA; 5 Present Address: International Institute of Anticancer Research, Kapandriti, 19014 Greece

Correction to: *Scientific Reports* 10.1038/s41598-017-18924-4, published online 11 January 2018

This article contains a typographical error in the Introduction section,

“This spectral region penetrates the skin deeper that UVB (280–320 nm), readily reaching the dermal collagen and elastic fibres^2^.”

should read:

“This spectral region penetrates the skin deeper than UVB (280–320 nm), readily reaching the dermal collagen and elastic fibres^2^.”

Additionally, this Article contains errors in Reference 24, which is incorrectly given as:

Tewari, A., Lahmann, C., Sarkany, R., Bergemann, J. & Young, A. R. Human erythema and matrix metalloproteinase-1 mRNA induction, *in vivo*, share an action spectrum which suggests common chromophores. *Photochem. Photobiol. Sci*. **11**, 216–223 (2012).

The correct reference is listed below as ref. 1.
